# *Chlorella sorokiniana* KU.B2 microalga inhibits *Aedes aegypti* larval development

**DOI:** 10.1016/j.cris.2026.100125

**Published:** 2026-05-27

**Authors:** Baby Kyi Soe, Nuttha Sanevas, Jemisha Dudhat, Saowalak Kaewmee, Chonlada Mano, Jassada Saingamsook, Urassaya Pattanawong, Roongtawan Muangmoon, Catherine Walton, Padet Siriyasatien, George Dimopoulos, Narissara Jariyapan

**Affiliations:** a Center of Excellence in Vector Biology and Vector-Borne Disease, Department of Parasitology, Faculty of Medicine, Chulalongkorn University, Bangkok 10330, Thailand; b Special Research Incubator Unit for Cryptogamic Biodiversity and Climate Responses, Department of Botany, Faculty of Science, Kasetsart University, Bangkok 10900, Thailand; c Department of Medical and Public Health Secretary, College of Allied Health Sciences, Suan Sunandha Rajabhat University, Samut Songkhram 75000, Thailand; d Department of Fundamentals of Public Health, Faculty of Public Health, Burapha University, Chonburi 20131, Thailand; e Parasitology and Entomology Research Cluster (PERC), Department of Parasitology, Faculty of Medicine, Chiang Mai University, Chiang Mai 50200, Thailand; f School of Earth and Environmental Sciences, Faculty of Science and Engineering, University of Manchester, Manchester M13 9PL, United Kingdom; g W. Harry Feinstone Department of Molecular Microbiology and Immunology, Bloomberg School of Public Health, Johns Hopkins University, Baltimore, MD 21205, USA

**Keywords:** *Aedes aegypti*, Biological control, *Chlorella sorokiniana*, Larval development, Microalgae, Mosquito vector

## Abstract

Insecticide-resistant *Aedes aegypti* (Linnaeus) poses a growing global challenge, reducing the effectiveness of conventional chemical control and raising the need for sustainable alternatives. Targeting the larval stage remains a key strategy in mosquito management. Here, the effects of the microalga *Chlorella sorokiniana* (Shihira & R.W. Krauss) strain KU.B2 on larval development, pupation, adult emergence, and mortality of *Ae. aegypti* were investigated, together with gut ultrastructural and histological observations. Larvae were reared under five dietary regimes: (i) rabbit food suspension alone (10 mg/mL; control); (ii–iv) rabbit food suspension maintained at 10 mg/mL supplemented with 25%, 50%, or 75% (v/v) microalgal suspension; and (v) 100% microalgal suspension. Larvae exposed to microalgae showed significantly reduced growth compared with controls. While control larvae completed development within 10 days, pupation and adult emergence were delayed and reduced in the lower microalgal proportions (25% and 50%). Conversely, larvae exposed to higher microalgal proportions (75% and 100%) remained predominantly arrested at the L2 stage (day 30), with mortality rates of 89.5% and 100%, respectively. Ultrastructural and histological analyses revealed persistence of intact and partially degraded microalgal cells in the larval gut of selected groups over time suggesting that prolonged retention of microalgal materials within the gut lumen might be associated with developmental inhibition. Fractionation experiments performed using a selected microalgal supplementation proportion (62.5%) further showed that whole microalgae, disrupted cell materials, and soluble fractions were all associated with larval developmental inhibition. Overall, *C. sorokiniana* KU.B2 exhibits larval development-inhibitory effects against *Ae. aegypti* under these laboratory conditions.

## Highlights

• *Chlorella sorokiniana* KU.B2 inhibited larval development of *Ae. aegypti* under laboratory conditions.
• Microalgal exposure reduced larval growth, delayed pupation, and lowered adult emergence.
• High microalgal proportions (75%–100%) arrested most larvae at the L2 stage and increased mortality (up to 100%).
• Intact and degraded microalgal cells persisted in the larval gut over time.
• Whole cells, disrupted cell materials, and soluble fractions all impaired larval development.

## Graphical abstract

**Figure:**
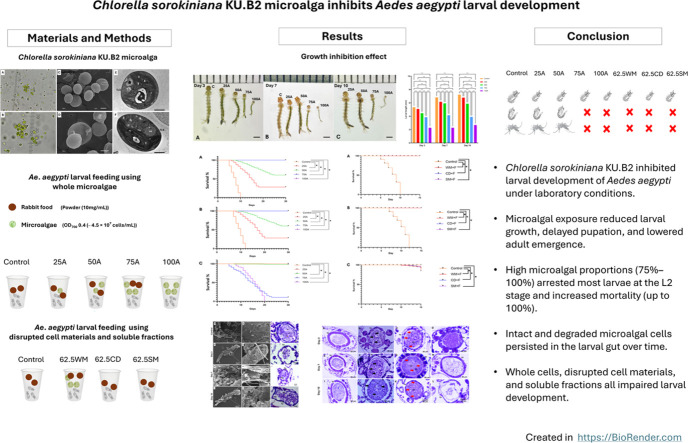

## 1 Introduction

*Aedes aegypti* is a primary vector for major arboviral diseases, including dengue, chikungunya, and Zika. The World Health Organization estimates that severe dengue affects roughly half a million people annually, with a significant mortality rate (Azeem et al., 2019) among children. In recent decades, the expansion of urban transmission cycles has intensified the risk of these diseases globally. In the absence of effective vaccines for most mosquito-borne viruses, vector control remains the primary strategy for disease prevention. Current management relies heavily on chemical insecticides (Tufan-Cetin and Cetin, 2023). However, the widespread overuse of these chemicals has driven the evolution of insecticide resistance in vector populations, creating an urgent need for alternative, sustainable control methods (Troppens and Neyts, 2024). Bioinsecticides, like microalgal extracts containing various active compounds, have been proposed to provide a safe, practical, and cost-effective alternative approach in biomedicine while supporting sustainable ecosystems (Costa et al., 2019). Bioinsecticides, particularly those derived from microalgae, offer a promising, eco-friendly avenue for vector management.

Microalgal extracts in mosquito breeding sites have attracted increasing interest from researchers and public health officials (Ahmad et al., 2001, 2004; Fei et al., 2021; Gil et al., 2021; Perumal et al., 2022; Gil et al., 2024). Mosquito larvae represent a critical target for biological control as this stage is confined to aquatic habitats and is the primary phase for feeding and growth (Jusoh et al., 2020). Microalgae are naturally present in mosquito breeding sites and serve as a food source; however, certain species can disrupt larval development through indigestibility or the production of toxic metabolites (Ahmad et al., 2001; Gil et al., 2024).

The genus *Chlorella* (Phylum: Chlorophyta) comprises unicellular green algae known for their adaptability and simple cellular structure (Krienitz et al., 2004; Safi et al., 2014). Green microalgae species can be isolated from diverse freshwater habitats such as ponds, lakes, and rivers and are distributed worldwide across Asia, Europe, and North America, where they are cultivated for applications including biofuel production and wastewater treatment (Becker, 2007; Mandal and Mallick, 2011). Specifically, *Chlorella sorokiniana* is of interest due to its rapid growth and suitability for commercial-scale cultivation (Chader et al., 2011; Kumar et al., 2014; Huesemann et al., 2016; Reyna-Martinez et al., 2018; Lomakool et al., 2023; Saraphol et al., 2024). Additionally, Sigamani et al. (2020) have identified n-hexadecanoic acid, oleic acid, and β-sitosterol in the active chloroform fractions of *Chlorella* sp., which have been reported to exhibit larvicidal effects against mosquito larvae.

Thailand remains endemic for several mosquito-borne diseases, including dengue hemorrhagic fever (DHF), dengue fever (DF), chikungunya, and Zika virus infection (Maneerattanasak et al., 2024). The exploration and utilization of indigenous biological resources are essential for developing sustainable and locally applicable vector control methods. *Chlorella sorokiniana* KU.B2, an indigenous microalgal strain isolated from Thailand, is recognized for its scalability and production of major bioactive metabolites such as 2-hexanol, n-hexadecanoic acid, and octadecanoic acid (Songserm et al., 2022; Kaeoboon et al., 2025). Despite this potential, the impact of the specific strain *C. sorokiniana* KU.B2 on *Ae. aegypti* has not been fully characterized. The objectives of this study were to investigate the effects of *C. sorokiniana* KU.B2 on larval development, pupation, adult emergence, and mortality of *Ae. aegypti*. In addition, gut ultrastructural and histological observations, together with fractionation experiments, were conducted to characterize treatment-associated differences in gut luminal contents and larval developmental responses.

## 2 Materials and methods

### 2.1 Microalgal cultivation

*Chlorella sorokiniana* strain KU.B2 used in this study was originally collected from freshwater samples in agricultural vegetable cultivation areas in Bang Yai Subdistrict, Nonthaburi Province, Thailand, in 2021. The strain was isolated and purified at the Department of Botany, Faculty of Science, Kasetsart University, according to the method described by Songserm et al. (2022), and has been maintained as a laboratory stock culture in the Department since 2021. The microalga was cultured in liquid tris-acetate-phosphate (TAP) medium in sterilized 250 mL glass bottles. Cultures were maintained at 30 ± 1 °C, pH 7, under cool white LED lighting (200 μmol photons m⁻² s) (Songserm et al., 2022).

### 2.2 Confirmation of microalgal species

Species identity was confirmed via amplification and sequencing of 18S rDNA and ITS2 gene fragments. Genomic DNA was extracted using the Qiagen DNeasy Plant Mini Kit (Qiagen, Germany). The 18S rDNA was amplified using primers 18SF (5′-GTCAGAGGTGAAATTCTTGGATTTA-3′) and 18SR (5′-AGGGCAGGGACGTAA TCAACG-3′) (Rasoul-Amini et al., 2009), and the ITS2 region was amplified with primers ITS-F1 (5′-GGAAGTAAAAGTCGTAACAAGG-3′) and ITS-R1 (5′-TCCTCCGCTTATT GATATGC-3′) (Baldwin, 1992). PCR products were cloned (TOPO TA Cloning Kit) and sequenced (Macrogen IncSeoul, South Korea). Sequences were analyzed using BLAST^n^, and reference sequences were retrieved from GenBank for phylogenetic analysis (https://www.ncbi.nlm.nih.gov/). Multiple sequence alignment was performed in MEGA software (version 11), and a maximum likelihood tree was constructed with IQ-TREE using 1000 bootstrap replicates. Phylogenetic relationships were visualized in FigTree (version 1.4.4).

The microalgal sample was morphologically identified using a light microscope (ECLIPSE E100, Nikon Corporation, Tokyo, Japan) based on taxonomic identification as previously described (Guiry, 2013; Nag Dasguspta et al., 2020; Songserm et al., 2022). Scanning electron microscopy (SEM) and transmission electron microscopy (TEM) analyses of the microalgal cells were performed as described below.

### 2.3 Mosquito rearing

A colony of *Ae. aegypti* (PMD strain) was established from larvae initially collected in Ban Pang Mai Daeng, Chiang Mai Province, Thailand. This colony has been maintained in the insectary of the Department of Parasitology at the Faculty of Medicine, Chiang Mai University, since 1997. Prior characterization revealed that the PMD strain exhibits resistance to DDT (dichlorodiphenyltrichloroethane) with mortality rates below 5% while demonstrating susceptibility to pyrethroid insecticides and lacking identifiable kdr mutations (Saingamsook et al., 2019). Filter papers containing the eggs were placed in 50-mL tubes, which were capped, sealed in plastic bags, and packed in a transport box. The egg parcels were shipped to the Department of Parasitology, Faculty of Medicine, Chulalongkorn University for further experiments. In the present study, synchronized 48-hr-old first-instar (L1) *Ae. aegypti* larvae were used in all experiments. Eggs were hatched under controlled laboratory conditions, and larvae of uniform age were selected prior to experimental exposure.

### 2.4 Preparation of larval diets

Microalgal cultures were centrifuged (1000 × g, 2 min) and washed three times with sterile distilled water. The pellet was resuspended in sterile distilled water to an Optical Density (OD)₇₀₀ of 0.4 (approx. 4.5 × 10⁷ cells/mL). Rabbit food pellets were ground into powder prior to diet preparation. Five diet treatments were prepared at the same final volume. Rabbit food was provided at a constant amount equivalent to 10 mg/mL in all groups except 100A, while only microalgal suspension was added at increasing proportions (v/v) relative to the final volume. Sterile distilled water was added as needed to achieve the same final volume across all treatments. Thereafter, five different diets were prepared: 1. Control: 100% rabbit food suspension; 2. 25A: rabbit food supplemented with 25% (v/v) microalgal suspension; 3. 50A: rabbit food supplemented with 50% (v/v) microalgal suspension; 4. 75A: rabbit food supplemented with 75% (v/v) microalgal suspension; 5. 100A: 100% microalgal suspension.

### 2.5 Assessment of larval development

To assess larval development, 150 L1 were assigned to each diet group and distributed into five plastic containers (30 larvae/container, 30 mL diet suspension/container). Larvae were maintained in multiple containers to minimize overcrowding and ensure more uniform access to the diet throughout the experimental period. On days 3, 7, and 10, larvae (n = 30 per group) were fixed in 70% ethanol and mounted in Hoyer’s medium. The mounted larvae were examined for morphological abnormalities under a stereomicroscope, and images were captured using a digital camera, an Olympus CX41RF light microscope (Tokyo, Japan). Larval length was measured and used to calculate the mean and standard deviation (SD) for each group at each time point (Gil et al., 2021) using ImageJ software (v.1.48). Three independent experiments were conducted.

Separate cohorts of 200 larvae per diet were monitored daily for 30 days following ingestion of the various diets. Each cohort was subdivided into five plastic containers (40 larvae/container, 40 mL diet suspension/container) to reduce larval crowding, promote uniform access to the diet, and facilitate daily monitoring and accurate counting of mortality, pupation, and adult emergence. These five containers together constituted a single experimental replicate. All experiments were performed in triplicate.

### 2.6 Preparation of disrupted cell materials (CD) and soluble fractions (SM) and experimental design to elucidate their effects on larval development

To better mimic the natural digestion of microalgae in the larval gut; therefore, mechanical grinding using a sterile plastic pestle was employed instead of ultrasonic homogenization. This approach was chosen because ultrasonic homogenization can generate excessive heat, which may alter or damage biological components and does not accurately reflect the action of microalgae in the larval gut. For the preparation of CD and SM, *C. sorokiniana* KU.B2 microalgae were harvested from high-density cultures (OD₇₀₀ = 2.4; approximately 2.7 × 10⁸ cells/mL). That were aliquoted into 1 mL microcentrifuge tubes (n = 100 tubes) for centrifugation at 4500 × rpm for 10 mins at 4 °C to collect microalgae. After removal of the supernatant the microalgae were washed three times with sterile distilled water at the same conditions. Harvested microalgae were next manually disrupted using a sterile plastic pestle for at least 3 mins until the suspension appeared visibly homogenized and placed on ice. The disrupted suspension was resuspended in 0.5 mL of sterile distilled water and centrifuged at 4500 × rpm for 10 mins at 4 °C to separate the CD and SM. Next, the supernatant containing SM was transferred to a sterile microcentrifuge tube and optical density (OD₇₀₀) was measured. The stock SM fraction was stored at 4 °C and used within 48 h. The stock was diluted with sterile distilled water to an approximate OD₇₀₀ of 0.4 before use. For the CD, the remaining pellets were pooled into a sterile microcentrifuge tube, washed three times with sterile distilled water through centrifugation at 4500 × rpm for 10 mins at 4 °C, and resuspended to an equivalent OD₇₀₀ of 0.4, matching the turbidity used for whole microalgae exposure treatments. The CD suspension was stored at 4 °C and used within 48 h. Aliquots of the CD suspension and SM fraction were examined microscopically to confirm cell disruption and the absence of intact cells (Fig. S1).

Based on preliminary results, 62.5% microalgal supplementation was selected as an intermediate proportion between 50% and 75%, at which larval development shifted from delayed progression to complete developmental arrest. This concentration was selected to evaluate the individual contributions of whole microalgal (WM) cells, CD, and SM to larval development under conditions where measurable effects on pupation, adult emergence, and mortality could be observed. Four experimental diets were prepared for each larval group. Group 1 received 100% rabbit food suspension (Control). Group 2 received rabbit food supplemented with 62.5% whole microalgal suspension (OD₇₀₀ 0.4) (WM+F), representing the combined CD and SM effects. Group 3 received rabbit food supplemented with 62.5% CD (OD₇₀₀ 0.4) (CD+F) to assess the contribution of CD in the absence of SM. Group 4 received rabbit food supplemented with 62.5% SM (OD₇₀₀ 0.4) (SM+F) to evaluate the effects of SM independent of CD. Larval development was assessed through pupation rate, adult emergence, and mortality. For each treatment, a total of 60 L1 were used. Larvae were allocated to each diet group and maintained in three plastic containers (20 larvae/container, 20 mL diet suspension/container) to reduce crowding and promote uniform feeding conditions and monitored daily for 15 days. Every two days, 200 µL of CD suspension (OD₇₀₀ 0.4) was added to the CD+F group, and 200 µL of SM suspension (OD₇₀₀ 0.4) was added to the SM+F group to maintain consistent exposure levels throughout the experimental period. This supplementation was designed to simulate continuous ingestion and digestion processes in the larval gut, as well as to preserve the activity of soluble fractions over time. All experiments were conducted in triplicate. Histological analyses were performed according to the protocol described in Section 2.8.

### 2.7 Electron microscopy (SEM and TEM)

The cultured microalgal sample and mosquito larvae were fixed in 2% glutaraldehyde for 12 h at 4 °C, washed twice with phosphate-buffered saline (PBS) (pH 7.4, Thermo Fisher Scientific, Waltham, USA), and subsequently fixed in 1% osmium tetroxide (OsO₄) at 25 °C. The samples were gradually dehydrated using an ethanol gradient (50%, 70%, 80%, 90%, and 100% v/v), dried with a critical point dryer (Leica model EM CPD300, Austria), and coated with osmium vapor using an osmium plasma coater (Blazers model SCD 040, Germany). After that the samples were examined using a scanning electron microscope (JSM-IT300, JEOL Ltd., Tokyo, Japan) operating at 100 kV as described (Kawasaki et al., 2020) at the Scientific and Technological Research Equipment Centre, Chulalongkorn University.

For TEM, the samples were prepared as described (Kawasaki et al., 2020). In brief, the microalgae sample was fixed overnight in 2% glutaraldehyde at 4 °C, then rinsed twice with PBS at ten-minute intervals, and subsequently post-fixed in 1% osmium tetroxide for 2 h. Following post-fixation, the specimens were rinsed twice with PBS, and dehydration was performed with a graded series of alcohol (30%, 50%, 70%, 80%, 90%, and 95% v/v). The specimens were then immersed in absolute alcohol for 12-hour periods to ensure complete dehydration and subsequently treated with acetone for 2 h. After that, the specimens were placed in resin mixtures with gradually increasing concentrations of Spurr’s resin (1:3 for 24 h, 1:1 for 24 h, and 3:1 for 24 h). Subsequently, the specimens were placed in pure resin twice for 3 h each to eliminate any remaining acetone. Finally, each sample was embedded in a plastic block filled with resin and incubated at 70 °C for 24 h before cutting the section (0.5 μm) using an ultramicrotome (Boeckeler®, Tucson, USA). Thereafter, the sections were stained with uranyl acetate and lead citrate and observed using a transmission electron microscope (ZEISS EM-10, Oberkochen, Germany) operating at 80 kV. Transmission electron microscopy of *C. sorokiniana* was performed at the Center of Medical Diagnostic Laboratories, Faculty of Medicine, Chulalongkorn University.

### 2.8 Histological analysis

To investigate histological alterations, the 3–5 larvae from the control and 100A on days 3, 7, and 10 were preserved in 4% formaldehyde at room temperature. Thereafter, dehydration was performed with a graded series of ethanol and subsequently embedded in paraffin using a tissue processor (INTELSINT S.R.L., Turin, Italy). Transverse sections of *Ae. aegypti* larvae were done using a rotary microtome (MICROM HM 340E, Thermo Fisher Scientific, Waltham, USA) and the sections were stained with Hematoxylin and Eosin (H&E). Finally, the stained samples were examined under a light microscope (Olympus U-TVO.63×C, Tokyo, Japan) and imaged using a digital camera (Olympus EP50, Tokyo, Japan). Histological analysis of larval gut was performed at the Department of Pathology, Faculty of Medicine, Chulalongkorn University.

### 2.9 Statistical analysis

Larval length data were obtained from three independent biological repeats. For each diet group and time point, the mean larval length from each biological repeat was used as the statistical unit. Prior to analysis, data were tested for normality using the Shapiro-Wilk test and homogeneity of variances using Levene’s test. Two-way ANOVA was used to assess the effects of diet, time, and their interaction, followed by Tukey’s post-hoc test for multiple comparisons. Pupation, adult emergence, and mortality data were analyzed using Kaplan-Meier time-to-event analysis, with group comparisons performed using the log-rank (Mantel-Cox) test in GraphPad Prism 10.4.0 (San Diego, CA, USA). For pupation, the event was defined as transition from larva to pupa, whereas for adult emergence, the event was defined as emergence from pupa to adult. Individuals that did not complete the respective developmental stage due to death or failure to develop by the end of the observation period were treated as censored observations.

### 2.10 Ethical statement

The study protocol was approved by the animal research ethics committee of Chulalongkorn University Animal Care and Use Protocol (CU-ACUP), Faculty of Medicine, Chulalongkorn University, Bangkok, Thailand (COA No 2491038). The research was initiated after receiving approval in accordance with the guidelines.

## 3 Results

### 3.1 Microalga species and morphological analysis

Amplification and sequencing of the 18S rDNA and ITS2 gene fragments were performed to confirm the species identity of the microalga. Two 18S rDNA sequences (816 bp) obtained in the present study exhibited 100% similarity to a *C. sorokiniana* isolate from Thailand (accession number KF444207). Phylogenetic analysis revealed that these sequences clustered with various *C. sorokiniana* strains and other *Chlorella* species retrieved from GenBank, specifically accession numbers KF444207, AB260897, AB240151, MH443351, MH619545, MK764925, KJ734869, MN103781, PP356659, PQ675342, JQ898145, LK021940, MH137235, and KR092112 (Fig. S2A). Regarding the ITS2 region, two sequences (861 bp) showed 98.96–99% similarity to a *C. sorokiniana* isolate from India (accession number JQ898145). Phylogenetic analysis demonstrated clustering with *C. sorokiniana* strains from diverse geographic regions deposited in GenBank (accession numbers JQ898145, MW776427, AB731602, MK764925, ON077056, KY303745, KU361155, and KJ676113) (Fig. S2B). Consequently, molecular and phylogenetic analyses confirmed that the microalgal strain employed in this study belongs to *C. sorokiniana*. The sequences generated, two 18S rDNA and two ITS2, were deposited in GenBank under accession numbers PX399778–PX399779 and PX399712–PX399713, respectively.

**Fig. 1:**
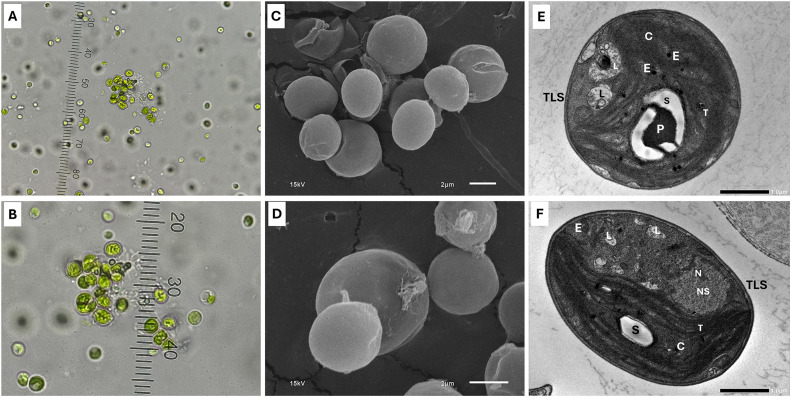
(A, B) Light micrographs of *C. sorokiniana* KU.B2, showing a smooth surface, a double-layered cell wall, and a bowl-shaped chloroplast with a single pyrenoid. (C, D) Scanning electron micrographs of *C. sorokiniana*, showing spherical to slightly ovoid cells with well-defined cell walls. The surface appears mostly smooth, with some cells exhibiting a granular texture. Cells are observed individually or in colonial aggregates. No flagella or external appendages are present, consistent with the non-motile nature of *Chlorella*. (E, F) Vegetative cells under TEM, the cell wall with trilaminar sheath (TLS) is seen. Cup-shaped chloroplast (C); Electron-dense reserve materials (E); Lipid bodies (L); Nucleolus (N); Nucleus (NS); Pyrenoid matrix (P); Starch grains (S); Thylakoids (T).

Morphological characterization via light microscopy revealed that the KU.B2 strain consists of spherical cells measuring 2–5 µm in diameter. The cells possess a smooth, non-flagellated cell wall enclosing a shallow, green, slightly bowl-shaped chloroplast with a single pyrenoid (Fig. 1A and B), a morphology that closely resembles descriptions of *C. sorokiniana* reported elsewhere. Scanning electron microscopy depicted spherical to slightly ovoid cells with well-defined cell walls. Cells appeared either individually or in aggregates (Fig. 1C and D), with diameters ranging from 2 to 5 µm. Consistent with the non-motile nature of the genus *Chlorella*, no flagella or appendages were observed. Transmission electron microscopy elucidated the cell wall ultrastructure, showing a trilaminar sheath (TLS) composed of an electron-translucent layer sandwiched between two electron-dense layers, the outer mature mother wall and an inner daughter wall, along with a fibrillar wall. Internally, the cells contained a cup-shaped chloroplast with aligned thylakoids, a central pyrenoid, electron-lucent starch grains, a distinct nucleus, and lipid bodies. The cytoplasm contained electron-dense reserve materials and showed no evidence of vesiculation (Fig. 1E and F).

### 3.2 Effect of C. sorokiniana KU.B2 microalga on larval development of *Ae. aegypti*

**Fig. 2:**
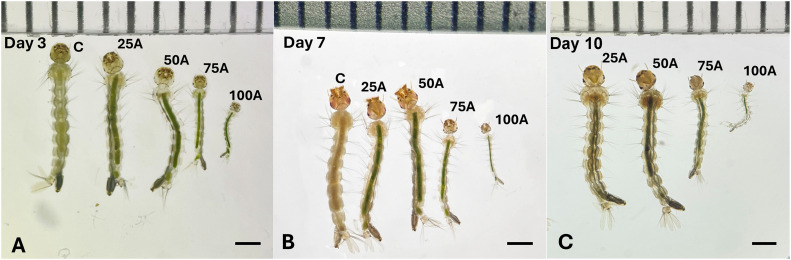
Representative *Ae. aegypti* larvae on (A) day 3, (B) day 7, and (C) day 10 that fed on control diet (C), mixed 25% algal suspension and 75% rabbit food (25A), mixed 50% algal suspension and 50% rabbit food (50A), mixed 75% algal suspension and 25% rabbit food (75A), and 100% algal suspension (100A). Scale bar: 1 mm.

**Fig. 3:**
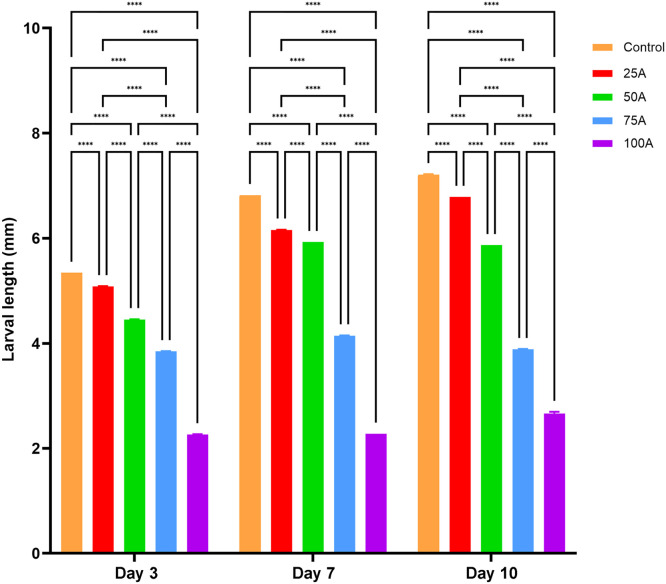
Larval growth of *Ae. aegypti* was assessed using body length (mm) as the outcome measure. Colored boxes represent different dietary treatment, and vertical bars indicate standard errors. Differences in larval development across diets were analyzed using two-way ANOVA, followed by Tukey’s multiple comparisons test. Larval length was measured under five dietary treatments on day 3, 7, and 10. Statistical significance was defined as ****p < 0.0001, reflecting significant variation in larval length between dietary groups.

Larval body length was measured on days 3, 7, and 10 to assess development (Fig. 2), revealing significant variations across dietary treatments and time points. Larvae in the 100A group exhibited the most limited developmental progression throughout the experiment, with significantly shorter body lengths than the control group on days 3 (2.26 ± 0.18 mm vs. 5.34 ± 0.34 mm) and 7 (2.28 ± 0.11 mm vs. 6.82 ± 0.41 mm). By day 10, control larvae had developed to the adult stage, whereas larvae in the 75A and 100A groups remained at the L2 stage, with body lengths of 3.89 ± 0.43 mm and 2.70 ± 0.21 mm, respectively. The 50A group also showed reduced larval growth compared with the control on days 3 (4.45 ± 0.27 mm) and 7 (5.93 ± 0.27 mm), while the 25A group displayed development comparable to the control on day 3 (5.08 ± 0.38 mm) but significantly reduced body length at days 7 (6.16 ± 0.33 mm) and 10 (6.79 ± 0.24 mm). Two-way ANOVA revealed significant effects of diet, time, and the interaction between diet and time on larval length (P < 0.0001). Tukey’s post hoc test further confirmed significant differences among dietary groups at each assessed time point (Fig. 3).

### 3.3 Effect of C. sorokiniana KU.B2 microalga on pupation, adult emergence, and mortality of *Ae. aegypti*

**Fig. 4:**
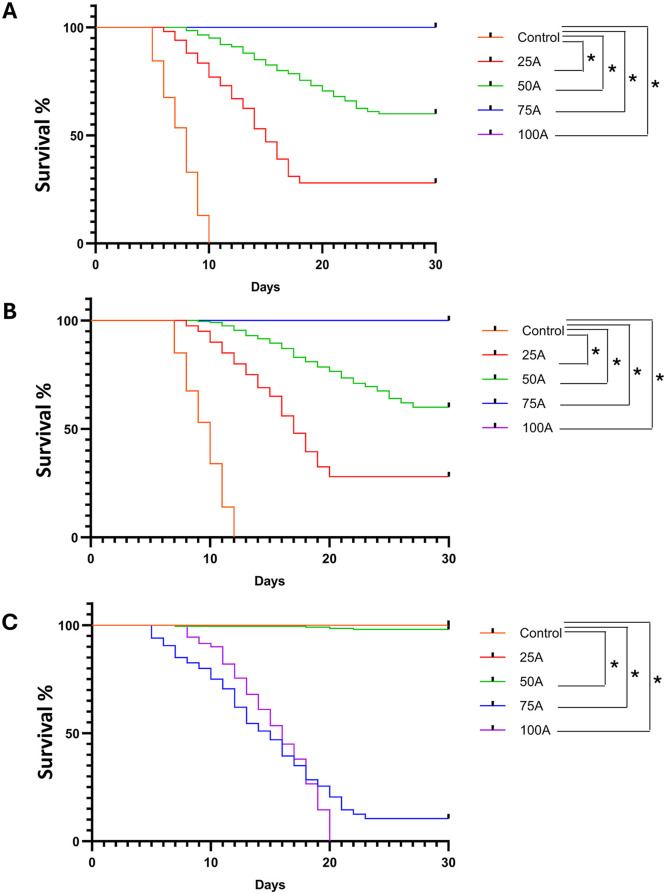
Effects of *C. sorokiniana* KU.B2 microalgae on the cumulative rates of pupation, adult emergence, and mortality of *Ae. aegypti* larvae under five dietary treatments. The Kaplan-Meier survival curves illustrate survival probabilities across the five dietary treatments. Panels show (A) survival probability to pupation, (B) survival probability to adult emergence, and (C) survival probability to mortality. Statistical significance was defined as *p < 0.05 using the Log-rank (Mantel-Cox) Test.

Pupation, adult emergence, and mortality of *Ae. aegypti* larvae were monitored from day 0 to day 30 across the five dietary groups (Fig. 4, Tables S1-S6). In the control group, 100% of larvae successfully pupated by day 10. The 25A and 50A diets also supported pupation, although development was delayed; pupation initiated on day 6 for the 25A group and day 8 for the 50A group, concluding on days 18 and 25, respectively. Despite this delay, pupation rates reached 72% in the 25A group and 40% in the 50A group. In contrast, no pupation was observed in larvae fed with the 75A or 100A diets throughout the 30-day observation period (Table S1). Larvae fed with 75A and 100A were arrested at the L2 stage. Kaplan-Meier survival analysis, followed by the log-rank (Mantel-Cox) test, revealed significant differences in pupation kinetics between the control and each diet group (Fig. 4A, Table S2).

Larvae in the control group exhibited normal development, with all individuals reaching the adult stage by day 12. Adult emergence in the 25A group followed a slower trajectory, reaching 72% by day 20. Similarly, emergence in the 50A group peaked at 40% by day 27. No adult emergence was recorded for the 75A and 100A groups (Table S3). All pupae that successfully emerged across the diet groups developed into morphologically normal adults. Kaplan-Meier analysis of time to adult emergence, combined with pairwise comparisons, confirmed significant differences between the control and each diet treatment. The control group demonstrated the highest and most rapid adult emergence, whereas the 25A and 50A diets resulted in progressively reduced and delayed emergence. Adult emergence remained absent in the 75A and 100A groups throughout the experiment (Fig. 4B, Table S4).

Regarding mortality, no deaths were observed in the control and 25A diet groups across all developmental stages, whereas cumulative mortality increased significantly at higher microalgal concentrations. In the 50A group, mortality was low (2%), with larvae dying on days 18, 20, and 22. However, extremely high mortality rates were observed in the 75A and 100A groups (89.5% and 100%, respectively), with deaths recorded between day 5 and day 30 (Table S5). Kaplan-Meier survival analysis with pairwise comparisons demonstrated significant differences in larval survival across dietary treatments. The highest mortality occurred in the 75A and 100A groups, while the control and 25A groups maintained 100% survival. Only the 50A, 75A, and 100A groups displayed a decline in survival over time (Fig. 4C. Table S6).

### 3.4 Ultrastructural and histological analyses of *Ae. aegypti* larval guts on days 3, 7, and 10

**Fig. 5:**
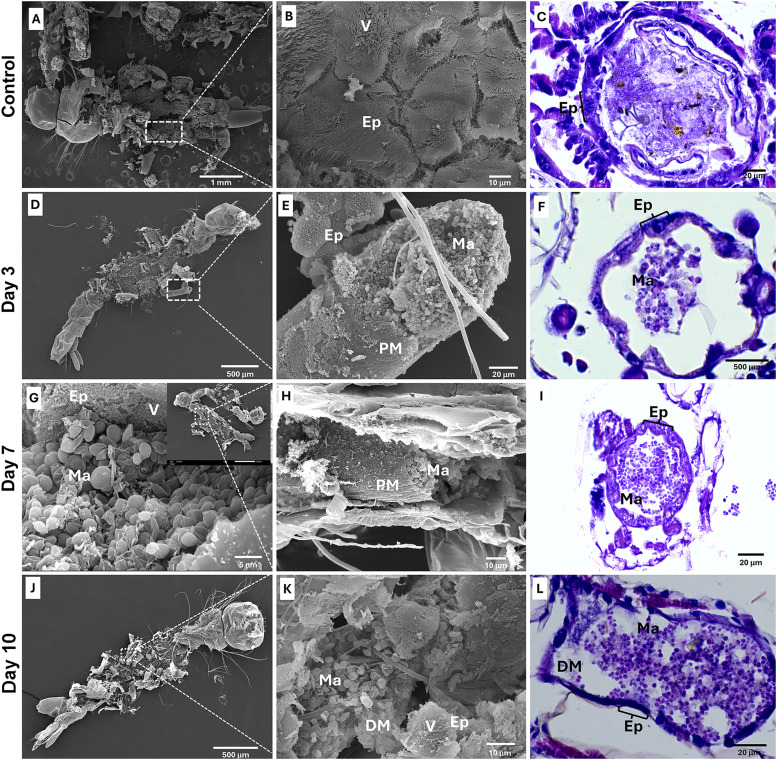
Ultrastructural (A, B, D, E, G, H, J, K) and histopathological (C, F, I, L) analyses in *Ae. aegypti* larval guts feeding on (A-C) control and (D-L) 100A diets. Observations on day 3 (D, E, F); day 7 (G, H, I); and day 10 (J, K, L). Disrupted materials (DM); Epithelial cells (Ep); Microalgae (Ma); Peritrophic membrane (PM); Villi (V).

Ultrastructural and histological analyses were conducted on larval guts at days 3, 7, and 10 to observe gut morphology and the presence of microalgae within the gut in the 100A group. Larvae fed the control diet exhibited normal gut ultrastructure and the peritrophic membrane throughout the experiment (up to day 10); likewise, the gut epithelium displayed normal morphology (Fig. 5A and B). Consistent with these ultrastructural observations, histological sections confirmed preserved tissue architecture and complete digestion in control larvae by day 10 (Fig. 5C). In contrast, in the 100A group, on days 3 and 7, numerous intact microalgal cells and some partially disrupted cells were visible within the gut lumen (Fig. 5D–I). Epithelial cells were structurally intact, and villi appeared with a well-defined organization (Fig. 5I). By day 10, both intact and degraded microalgal cells were observed in the larval guts (Figs. 5J–L). No obvious histological or ultrastructural changes in gut epithelial cells were identified. Nonetheless, these observations were qualitative rather than grounded on quantitative measurements.

### 3.5 Effect of disrupted cell materials and soluble fractions on pupation, adult emergence, and mortality of *Ae. aegypti*

**Fig. 6:**
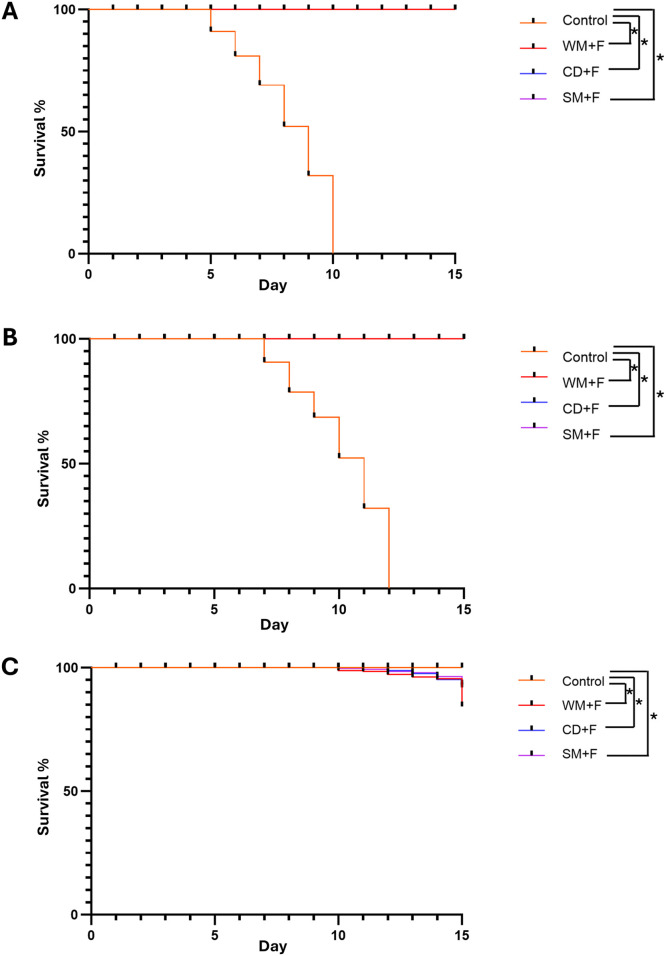
Effects of *C. sorokiniana* KU.B2-derived soluble metabolites and cell debris on the cumulative rates of pupation, adult emergence, and mortality of *Ae. aegypti* larvae under four dietary treatments. The Kaplan-Meier survival curves illustrate survival probabilities across the four dietary treatments. Panels show (A) survival probability to pupation, (B) survival probability to adult emergence, and (C) survival probability to mortality. Statistical significance was defined as *p < 0.05 using the Log-rank (Mantel-Cox) Test.

To further investigate whether larval developmental inhibition was associated with particulate or soluble microalgal fractions, the effects of WM, CD, and SM on larval development, pupation, adult emergence, and mortality of *Ae. aegypti* were monitored for 15 days across four dietary groups, including control, WM+F, CD+F, and SM+F. In the control group, all larvae pupated from day 5 to day 10 and reached adulthood by day 12. No pupation was observed with the remaining three diets, and most larvae arrested at the L2 stage (>99% in groups CD+F and SM+F; >90% in group WM+F) until day 15 (Fig. 6A and B, Tables S7-S10). No mortality was observed in the control group, whereas mortality had occurred between days 10 and 15 in the WM+F, CD+F, and SM+F diet groups. Kaplan-Meier survival analysis demonstrated significant differences in larval mortality patterns compared to the control group (Fig. 6C, Tables S11–S12).

**Fig. 7:**
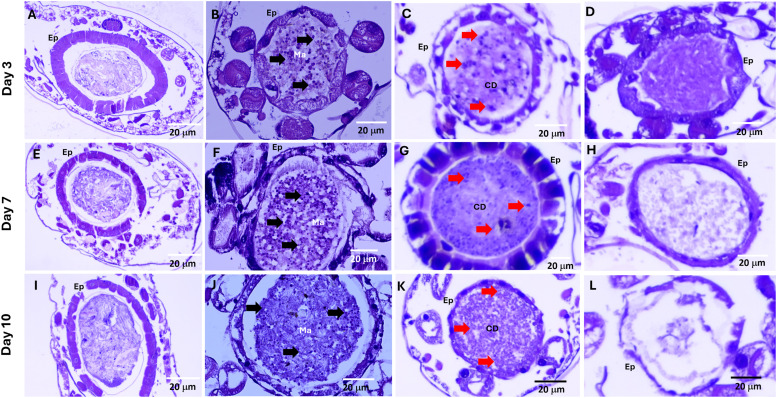
Histological analysis of *Ae. aegypti* larval gut fed on control and microalgal-supplemented diets on days 3 (A–D), 7 (E–H), and 10 (I–L). Day 3: (A) Control diet, (B) WM+F diet, (C) CD+F diet, and (D) SM+F diet. Day 7: (E) Control diet, (F) WM+F diet, (G) CD+F diet, and (H) SM+F diet. Day 10: (I) Control diet, (J) WM+F diet, (K) CD+F diet, and (L) SM+F diet. Disrupted cell materials (CD); Epithelial cells (Ep); Microalgae (Ma). Black arrow shows intact microalgal cells, whereas red arrow shows microalgal cell debris.

Histological analyses revealed distinct gut luminal contents among larvae fed different diets. On day 3, larvae fed with control diet showed gut contents without visible particulate materials (Fig. 7A). Intact microalgal cells were observed within the gut lumen of the WM+F group (Fig. 7B), while clusters of disrupted cell materials were evident in the CD+F group (Fig. 7C). The SM+F group displayed gut contents comparable in appearance to those observed in the control group, without visible particulate accumulation (Fig. 7D). By day 7, the control group continued to show homogeneous gut contents (Fig. 7E). In contrast, intact microalgal cells remained visible in the gut lumen of the WM+F group, while disrupted cell material aggregates persisted in the CD+F group (Fig. 7F, G). No visible accumulation of particulate material was observed in the SM+F group (Fig. 7H). By day 10, larvae in the control group maintained similar gut morphology and luminal appearance (Fig. 7I). In contrast, substantial luminal accumulation of intact microalgal biomass in the WM+F group and disrupted cell materials in the CD+F group was observed (Fig. 7J, K). Larvae fed the SM+F diet similarly showed no visible luminal accumulation (Fig. 7L). Despite these differences in gut luminal contents, larvae from the WM+F, CD+F, and SM+F groups remained developmentally arrested at the L2 stage.

## 4 Discussion

The constraints of conventional insecticides, including pest chemical resistance and negative environmental effects, have driven interest in alternative sustainable and environmentally friendly vector control methods (Troppens and Neyts, 2024). Consequently, microalgae have gained interest as a potential biological resource for the development of novel bioinsecticides (Gil et al., 2024; Izydorczyk et al., 2025). In the present study, strain KU.B2 was identified as *C. sorokiniana* based on phylogenetic and morphological data for the first time.

We also observed that *C. sorokiniana* KU.B2 affected the development of *Ae. aegypti* larvae. Although larvae on a regular diet developed normally, increasing proportions of microalgal biomass were associated with delayed development, diminished size, and complete developmental collapse during early instars at elevated concentrations. These observations are consistent with previous studies reporting that some microalgae are associated with impaired larval development. For examples, *Chlorococcum* and *Scenedesmus* species, are poorly digested, leading to inhibited larval growth during early developmental stages (Ahmad et al., 2004; Díaz-Nieto et al., 2016). Similarly, nutritional limitation or altered food quality has also been shown to negatively affect development in *Ae. aegypti* (Zeller and Koella, 2016).

The persistence of intact and partially degraded *C. sorokiniana* cells in the larval gut over time suggests prolonged retention of microalgal materials within the larval gut lumen, which might be associated with developmental inhibition. However, the biological significance of this retention remains unclear, as no quantitative or functional analyses of digestion or gut physiology were performed. Similar observations have been reported in previous studies showing that *Ae. aegypti* larvae exhibit limited utilization of several microalgal taxa, including *Chlorella* spp. (He et al., 2016; Souza et al., 2019). This phenomenon may be related to the biochemical structure of the *C. sorokiniana* cell wall, which is abundant in glucosamine-based polymers and rhamnose polysaccharides that impart stiffness and resistance to enzymatic degradation (Takeda, 1991; Russell, 1995). However, nutrient assimilation, digestive efficiency, and nutrient absorption were not directly evaluated in the present study. Therefore, the observed persistence of microalgal materials cannot be interpreted as definitive evidence of impaired digestion or reduced nutrient uptake. Notably, the ultrastructural and histological images from this study did not reveal clear evidence of damage to the gut epithelial cells. Also, whether ingestion of microalgal materials affects gut epithelial integrity could not be conclusively determined from the current observations. Further ultrastructural analyses, such as transmission electron microscopy, together with biochemical or enzymatic assays, would be required to clarify these potential effects and better understand the mechanisms underlying larval growth inhibition. In the case of the 100A treatment, nutritional limitation should also be considered, since larvae in this group received only microalgal biomass without rabbit food supplementation. Therefore, developmental arrest and mortality under this condition may partly reflect inadequate nutritional support in addition to a direct microalgal effect alone.

The effects of CD and SM fractions on larval development were further investigated in this study. Across all microalgae-supplemented diets (WM+F, CD+F, and SM+F), larvae failed to pupate and remained predominantly arrested at the L2 stage until day 15, in contrast to the control group, which showed normal development to adulthood. Histological observations revealed distinct gut luminal patterns among treatments. Intact microalgal cells persisted in the gut lumen of larvae fed the WM+F diet, whereas particulate, disrupted cell materials accumulated in the CD+F group over time. In contrast, larvae fed the SM+F diet showed no visible luminal accumulation but remained developmentally arrested at the L2 stage. As the CD fraction was generated from mechanically disrupted microalgal biomass, it likely contained particulate materials derived from disrupted cellular structures, including cell wall-associated components, such as glucosamine-based polymers and rhamnose-rich polysaccharides (Takeda, 1991; Russell, 1995). In contrast, the SM fraction represented the soluble portion obtained following cell disruption. However, the chemical composition of either fraction was not characterized in the present study. Therefore, although both fractions were associated with larval developmental inhibition, the specific components contributing to these effects remain unclear. Taken together, these results suggest that both CD and SM fractions of *C. sorokiniana* KU.B2 were associated with larval developmental inhibition, although the mechanisms underlying these effects were not directly investigated in the present study. Further physiological, biochemical, and molecular analyses will be required to determine how these fractions influence larval development and survival.

*Chlorella sorokiniana* KU.B2 has been reported to produce a diverse range of bioactive metabolites with antioxidant properties, including compounds such as 2-hexanol, n-hexadecanoic acid, and octadecanoic acid (Songserm et al., 2022; Kaeoboon et al., 2025). GC–MS analysis of the methanolic extract revealed the presence of n-hexadecanoic acid, oleic acid, and β-sitosterol in *Chlorella* sp., metabolites previously associated with larvicidal activity against *Ae. aegypti* (Sigamani et al., 2020). Moreover, hexadecanoic acid has been demonstrated to exert significant bioactivity against *Anopheles stephensi* mosquito larvae, causing mortality in third-instar larvae and pupae at relatively low concentrations (Hassan and Jebanesan, 2022). Several studies have shown that algae can exert diverse effects on mosquito larvae depending on species, composition, and exposure conditions. Some microalgae serve as nutritional resources that support larval growth and development, whereas others impair development or cause mortality through inhibitory biological effects (Ahmad et al., 2004; Tufan-Cetin and Cetin, 2023; Gil et al., 2024).

Several non-exclusive mechanisms have been proposed to explain algal-mediated growth suppression or mortality in mosquito larvae including nutritional limitation and lethal toxicity by bioactive metabolites. One possible mechanism involves poor digestibility and physical interference which may reduce nutrient acquisition and ultimately result in starvation or development arrest. Larvae are unable to efficiently digest or assimilate nutrients from algal biomass, resulting in delayed growth, reduced body size, and developmental arrest. For physical interference, persistent intact cells or accumulated particulate materials may occupy gut lumen space, potentially limiting ingestion or processing of other food particles. This has been suggested for poorly digestible taxa such as *Chlorella, Scenedesmus*, and other chlorophytes with rigid cell walls (Ahmad et al., 2004; Voigt et al., 2014; He et al., 2016). A second proposed mechanism may involve chemical or toxic effects mediated by algal metabolites or cell-associated compounds. Both microalgae and macroalgae have been reported to contain bioactive molecules, including fatty acid derivatives, phenolics, terpenoids, sterols, and other secondary metabolites with insecticidal or growth-inhibitory activity against mosquito larvae (Raj et al., 2017; Sigamani et al., 2020; Rashed et al., 2024; Refaay et al., 2025).

Although *Chlorella* species are widely regarded as safe and are commonly used in nutritional and aquaculture applications, our findings highlight their context-dependent bioactivity, particularly against mosquito larvae. However, translation of these findings into practical mosquito control strategies requires further evaluation beyond laboratory conditions. Important operational considerations include the environmental persistence and stability of active microalgal fractions under natural breeding conditions, particularly in relation to temperature fluctuations, UV exposure, and microbial degradation, all of which may influence larvicidal performance over time. In addition, the scalability and cost-effectiveness of biomass production, fractionation, and formulation must be assessed to determine feasibility for large-scale implementation. Although *Chlorella* microalgae are generally considered safe, potential effects on non-target aquatic organisms should also be carefully evaluated prior to field deployment, especially given the observed bioactivity of disrupted and soluble fractions. Furthermore, efficacy under natural breeding-site conditions may differ substantially from laboratory settings due to variation in water quality, organic content, larval density, and availability of competing food sources. Therefore, additional semi-field and field studies are needed to assess environmental safety, formulation stability, application strategies, and overall operational applicability for mosquito control.

## 5 Conclusion

In the present study, the indigenous microalgal strain KU.B2 was identified as *C. sorokiniana* based on phylogenetic and morphological analyses. Our findings showed that *C. sorokiniana* KU.B2 inhibited the development of *Ae. aegypti* larvae in a dose-dependent manner. Lower microalgal proportions (25% and 50% v/v) delayed and reduced pupation, whereas higher proportions (75% and 100% v/v) caused persistent developmental arrest at the L2 stage, accompanied by mortality rates of 89.5% and 100%, respectively. Using a selected microalgal proportion (62.5% v/v), fractionation experiments further showed that CD and SM fractions of *C. sorokiniana* were associated with larval developmental inhibition, although the underlying mechanisms were not directly investigated in this study. Further studies are required to assess environmental safety, formulation stability, and practical application in semi-field and field studies prior to considering this microalgal strain as a potential eco-friendly biological control agent in integrated vector management systems.

## CRediT authorship contribution statement

Conceptualization: NJ, BKS. Data curation: NJ, BKS, NS, SK. Formal analysis: NJ, BKS, JD, JS. Funding Acquisition: NJ. Investigation: BKS, JD, SK, CM, NJ. Methodology: NJ, BKS, JS, NS. Project administration: NJ. Resources: NS, JS. Supervision: NJ, PS, CW. Validation: NJ. Visualization: BKS, NJ, SK, UP, RM. Writing-original draft: BKS, NJ. Writing-review and editing: NJ, BKS, WC, PS, GD.

## Funding

This research project was funded by the National Research Council of Thailand (NRCT) and Chulalongkorn University (grant number N42A660641), the Research Grant Funding from the Ratchadaphiseksomphot Fund, Faculty of Medicine, Chulalongkorn University (Grant No RA68/036), and the Second Century Fund (C2F), Chulalongkorn University, to NJ for BKS.

## Data availability

Data supporting the conclusions of this article are included within the article and the sequences were deposited in the GenBank under accession numbers PX399778 - PX399779 for 18S and accession numbers PX399712 - PX399713 for ITS.

## Declaration of competing interest

The authors declare that they have no known competing financial interests or personal relationships that could have appeared to influence the work reported in this paper.

## Appendix Supplementary materials

Image, application 1

Image, application 2
