# Supplementary material for: Chlorella sorokiniana KU.B2 microalga inhibits Aedes aegypti larval development
Source: Curr Res Insect Sci. 2026 May 27;9:100125. doi: 10.1016/j.cris.2026.100125 (PMC13253192; doi:10.1016/j.cris.2026.100125)

**Fig. S1.** Preparation of microalgal soluble fractions (SM) and disrupted cell materials (CD). (A) Macroscopic appearance of SM (upper) and CD (lower) fractions. (B) SM fraction showing no intact cells. (C) CD fraction showing particulate cellular debris. Scale bars in µm.


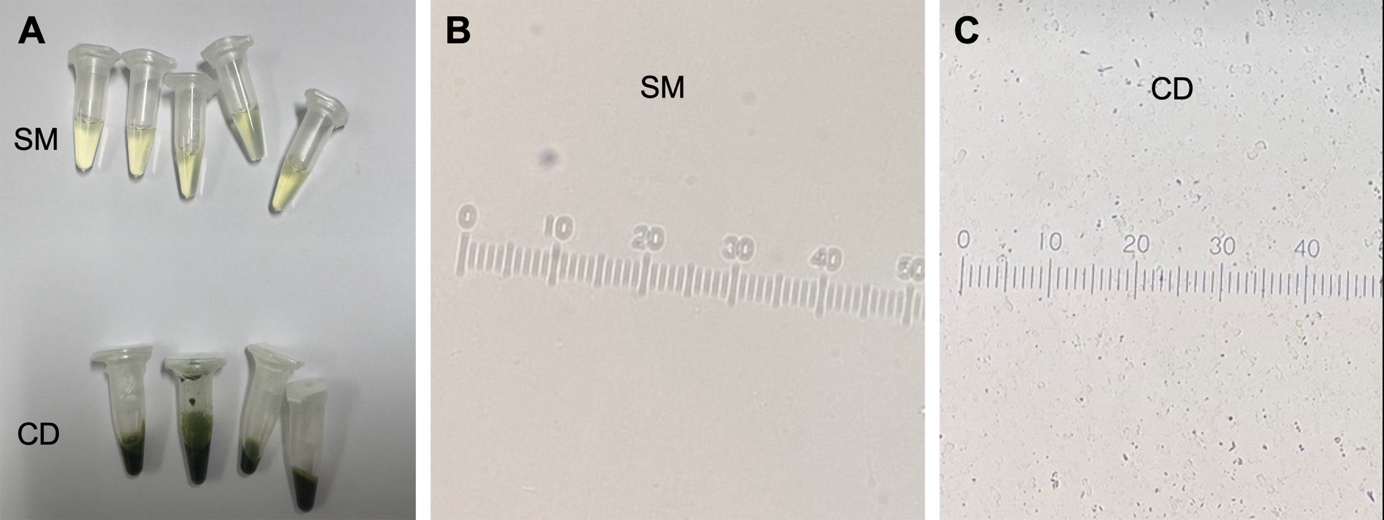


**Fig. S2.** (A) A phylogenetic tree generated using two samples of 18S rDNA sequences of the isolated microalgal strain from the present study together with 12 reference sequences retrieved from GenBank. A total of 14 sequences of genus *Chlorella* were analyzed using the maximum likelihood method with the JC model under 1,000 bootstrap replicates using IQTree software. Tree with branch lengths indicating number of substitutions per site. The sequences from the present study are written in red. (B) A phylogenetic tree generated using two samples of ITS sequences of the isolated microalgal strain from the present study together with 12 reference sequences retrieved from GenBank. A total of 14 sequences were analyzed using the maximum likelihood method with the JC model under 1,000 bootstrap replicates using IQTree software. Tree with branch lengths indicating number of substitutions per site. The sequences from the present study are written in red.


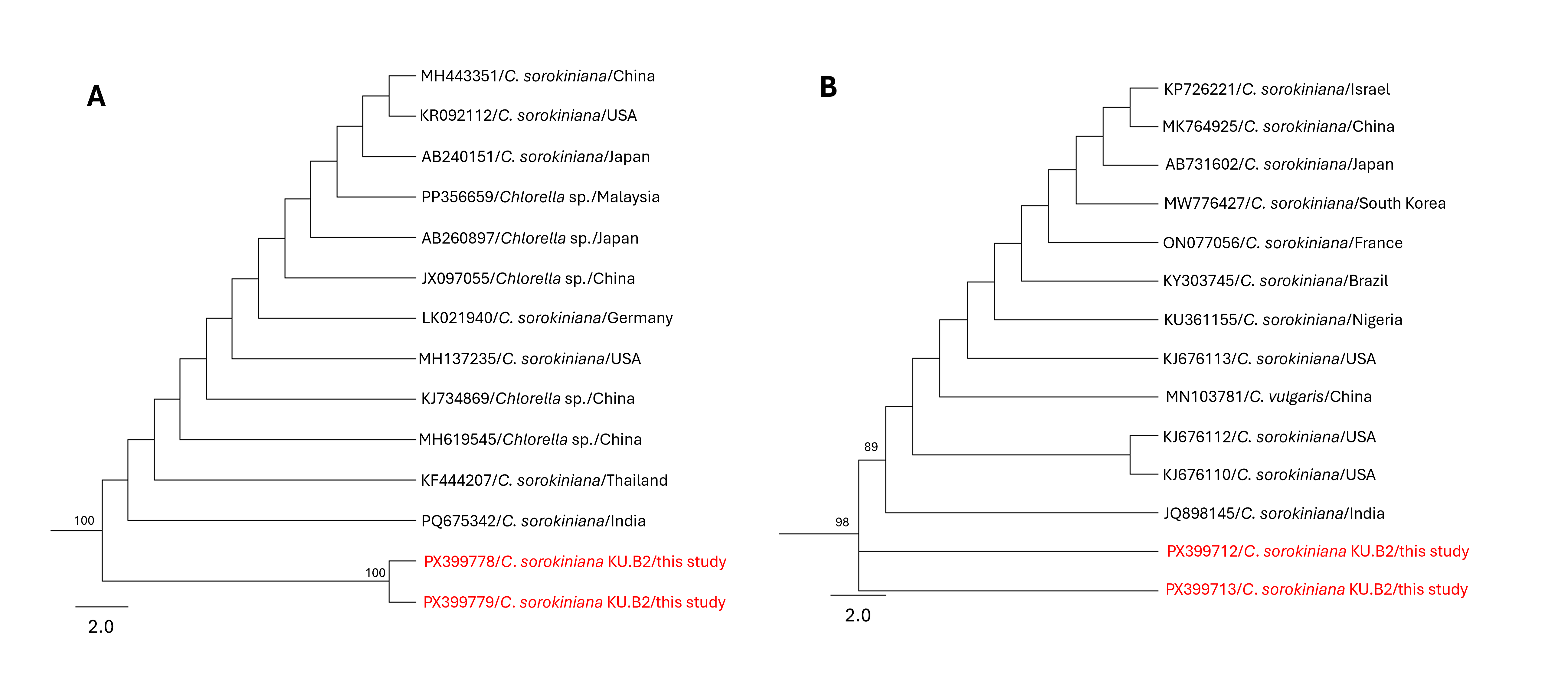

Supplement: Supplementary file 1 [file mmc1.docx]
