# Supplementary material for: Chlorella sorokiniana KU.B2 microalga inhibits Aedes aegypti larval development
Source: Curr Res Insect Sci. 2026 May 27;9:100125. doi: 10.1016/j.cris.2026.100125 (PMC13253192; doi:10.1016/j.cris.2026.100125)
Supplement: Supplementary file 2 [file mmc2.docx]

**Table S1.** Cumulative pupation rates of larvae fed on different diets using whole microalgae with different proportions from day 1 to day 30 are shown in Mean ± SD.

| Day | Pupation rate (Mean ± SD) | | | | |
| --- | --- | --- | --- | --- | --- |
|  | Control | 25A | 50A | 75A | 100A |
| 1 | 0.0 ± 0.0 | 0.0 ± 0.0 | 0.0 ± 0.0 | 0.0 ± 0.0 | 0.0 ± 0.0 |
| 2 | 0.0 ± 0.0 | 0.0 ± 0.0 | 0.0 ± 0.0 | 0.0 ± 0.0 | 0.0 ± 0.0 |
| 3 | 0.0 ± 0.0 | 0.0 ± 0.0 | 0.0 ± 0.0 | 0.0 ± 0.0 | 0.0 ± 0.0 |
| 4 | 0.0 ± 0.0 | 0.0 ± 0.0 | 0.0 ± 0.0 | 0.0 ± 0.0 | 0.0 ± 0.0 |
| 5 | 16.3 ± 0.76 | 0.0 ± 0.0 | 0.0 ± 0.0 | 0.0 ± 0.0 | 0.0 ± 0.0 |
| 6 | 31.8 ± 1.53 | 1.3 ± 1.16 | 0.0 ± 0.0 | 0.0 ± 0.0 | 0.0 ± 0.0 |
| 7 | 47.0 ± 1.00 | 5.2 ± 1.44 | 0.0 ± 0.0 | 0.0 ± 0.0 | 0.0 ± 0.0 |
| 8 | 66.8 ± 1.04 | 11.3 ± 1.16 | 1.5 ± 0.00 | 0.0 ± 0.0 | 0.0 ± 0.0 |
| 9 | 86.8 ± 1.60 | 15.7 ± 1.44 | 4.0 ± 0.87 | 0.0 ± 0.0 | 0.0 ± 0.0 |
| 10 | 100 ± 0.58 | 21.8 ± 1.60 | 5.5 ± 0.87 | 0.0 ± 0.0 | 0.0 ± 0.0 |
| 11 | 0.0 ± 0.0 | 26.2 ± 1.04 | 9.3 ± 2.31 | 0.0 ± 0.0 | 0.0 ± 0.0 |
| 12 | 0.0 ± 0.0 | 32.3 ± 1.26 | 10.8 ± 3.18 | 0.0 ± 0.0 | 0.0 ± 0.0 |
| 13 | 0.0 ± 0.0 | 36.8 ± 2.52 | 13.3 ± 2.31 | 0.0 ± 0.0 | 0.0 ± 0.0 |
| 14 | 0.0 ± 0.0 | 46.0 ± 2.00 | 16.3 ± 2.31 | 0.0 ± 0.0 | 0.0 ± 0.0 |
| 15 | 0.0 ± 0.0 | 52.7 ± 3.06 | 18.3 ± 1.89 | 0.0 ± 0.0 | 0.0 ± 0.0 |
| 16 | 0.0 ± 0.0 | 59.0 ± 1.00 | 20.3 ± 2.02 | 0.0 ± 0.0 | 0.0 ± 0.0 |
| 17 | 0.0 ± 0.0 | 66.7 ± 1.16 | 22.5 ± 3.12 | 0.0 ± 0.0 | 0.0 ± 0.0 |
| 18 | 0.0 ± 0.0 | 70.8 ± 1.76 | 25.3 ± 2.84 | 0.0 ± 0.0 | 0.0 ± 0.0 |
| 19 | 0.0 ± 0.0 | 0.0 ± 0.0 | 27.7 ± 2.57 | 0.0 ± 0.0 | 0.0 ± 0.0 |
| 20 | 0.0 ± 0.0 | 0.0 ± 0.0 | 30.3 ± 2.36 | 0.0 ± 0.0 | 0.0 ± 0.0 |
| 21 | 0.0 ± 0.0 | 0.0 ± 0.0 | 32.5 ± 1.80 | 0.0 ± 0.0 | 0.0 ± 0.0 |
| 22 | 0.0 ± 0.0 | 0.0 ± 0.0 | 34.5 ± 1.80 | 0.0 ± 0.0 | 0.0 ± 0.0 |
| 23 | 0.0 ± 0.0 | 0.0 ± 0.0 | 36.2 ± 2.08 | 0.0 ± 0.0 | 0.0 ± 0.0 |
| 24 | 0.0 ± 0.0 | 0.0 ± 0.0 | 38.2 ± 1.61 | 0.0 ± 0.0 | 0.0 ± 0.0 |
| 25 | 0.0 ± 0.0 | 0.0 ± 0.0 | 40.2 ± 1.61 | 0.0 ± 0.0 | 0.0 ± 0.0 |
| 26 | 0.0 ± 0.0 | 0.0 ± 0.0 | 0.0 ± 0.0 | 0.0 ± 0.0 | 0.0 ± 0.0 |
| 27 | 0.0 ± 0.0 | 0.0 ± 0.0 | 0.0 ± 0.0 | 0.0 ± 0.0 | 0.0 ± 0.0 |
| 28 | 0.0 ± 0.0 | 0.0 ± 0.0 | 0.0 ± 0.0 | 0.0 ± 0.0 | 0.0 ± 0.0 |
| 29 | 0.0 ± 0.0 | 0.0 ± 0.0 | 0.0 ± 0.0 | 0.0 ± 0.0 | 0.0 ± 0.0 |
| 30 | 0.0 ± 0.0 | 0.0 ± 0.0 | 0.0 ± 0.0 | 0.0 ± 0.0 | 0.0 ± 0.0 |

**Table S2.** P-values of pairwise comparisons of survival curves of pupation probability.

| **Comparison** | **Log-rank (Mantel-Cox) Test** | | |
| --- | --- | --- | --- |
|  | **χ2** | **df** | **p** |
| Control vs 25A | 292.1 | 1 | < 0.0001* |
| Control vs 50A | 417.0 | 1 | < 0.0001* |
| Control vs 75A | 449.5 | 1 | < 0.0001* |
| Control vs 100A | 449.5 | 1 | < 0.0001* |

*: Significance level p-value < 0.05

**Table S3.** Cumulative adult emergence rates of larvae fed on different diets using whole microalgae with different proportions from day 1 to day 30 are shown in Mean ± SD.

| Day | Adult emergence rate (Mean ± SD) | | | | |
| --- | --- | --- | --- | --- | --- |
|  | Control | 25A | 50A | 75A | 100A |
| 1 | 0.0 ± 0.0 | 0.0 ± 0.0 | 0.0 ± 0.0 | 0.0 ± 0.0 | 0.0 ± 0.0 |
| 2 | 0.0 ± 0.0 | 0.0 ± 0.0 | 0.0 ± 0.0 | 0.0 ± 0.0 | 0.0 ± 0.0 |
| 3 | 0.0 ± 0.0 | 0.0 ± 0.0 | 0.0 ± 0.0 | 0.0 ± 0.0 | 0.0 ± 0.0 |
| 4 | 0.0 ± 0.0 | 0.0 ± 0.0 | 0.0 ± 0.0 | 0.0 ± 0.0 | 0.0 ± 0.0 |
| 5 | 0.0 ± 0.0 | 0.0 ± 0.0 | 0.0 ± 0.0 | 0.0 ± 0.0 | 0.0 ± 0.0 |
| 6 | 0.0 ± 0.0 | 0.0 ± 0.0 | 0.0 ± 0.0 | 0.0 ± 0.0 | 0.0 ± 0.0 |
| 7 | 16.3 ± 1.26 | 0.0 ± 0.0 | 0.0 ± 0.0 | 0.0 ± 0.0 | 0.0 ± 0.0 |
| 8 | 35.0 ± 2.18 | 2.7 ± 0.29 | 0.0 ± 0.0 | 0.0 ± 0.0 | 0.0 ± 0.0 |
| 9 | 49.0 ± 1.73 | 6.0 ± 0.87 | 0.5 ± 0.0 | 0.0 ± 0.0 | 0.0 ± 0.0 |
| 10 | 67.2 ± 1.61 | 11.0 ± 0.87 | 1.0 ± 0.0 | 0.0 ± 0.0 | 0.0 ± 0.0 |
| 11 | 85.7 ± 1.04 | 16.0 ± 0.87 | 2.5 ± 0.0 | 0.0 ± 0.0 | 0.0 ± 0.0 |
| 12 | 100 ± 0.0 | 21.0 ± 1.32 | 4.2 ± 0.58 | 0.0 ± 0.0 | 0.0 ± 0.0 |
| 13 | 0.0 ± 0.0 | 26.2 ± 1.26 | 7.0 ± 0.0 | 0.0 ± 0.0 | 0.0 ± 0.0 |
| 14 | 0.0 ± 0.0 | 31.8 ± 1.44 | 8.7 ± 0.29 | 0.0 ± 0.0 | 0.0 ± 0.0 |
| 15 | 0.0 ± 0.0 | 36.2 ± 1.26 | 10.7 ± 0.29 | 0.0 ± 0.0 | 0.0 ± 0.0 |
| 16 | 0.0 ± 0.0 | 44.7 ± 0.58 | 13.3 ± 0.56 | 0.0 ± 0.0 | 0.0 ± 0.0 |
| 17 | 0.0 ± 0.0 | 52.8 ± 0.76 | 17.2 ± 0.29 | 0.0 ± 0.0 | 0.0 ± 0.0 |
| 18 | 0.0 ± 0.0 | 60.5 ± 1.0 | 19.2 ± 0.29 | 0.0 ± 0.0 | 0.0 ± 0.0 |
| 19 | 0.0 ± 0.0 | 67.3 ± 0.76 | 21.7 ± 0.29 | 0.0 ± 0.0 | 0.0 ± 0.0 |
| 20 | 0.0 ± 0.0 | 72.0 ± 0.0 | 23.7 ± 0.29 | 0.0 ± 0.0 | 0.0 ± 0.0 |
| 21 | 0.0 ± 0.0 | 0.0 ± 0.0 | 26.7 ± 0.29 | 0.0 ± 0.0 | 0.0 ± 0.0 |
| 22 | 0.0 ± 0.0 | 0.0 ± 0.0 | 29.2 ± 0.29 | 0.0 ± 0.0 | 0.0 ± 0.0 |
| 23 | 0.0 ± 0.0 | 0.0 ± 0.0 | 30.7 ± 0.29 | 0.0 ± 0.0 | 0.0 ± 0.0 |
| 24 | 0.0 ± 0.0 | 0.0 ± 0.0 | 32.7 ± 0.29 | 0.0 ± 0.0 | 0.0 ± 0.0 |
| 25 | 0.0 ± 0.0 | 0.0 ± 0.0 | 36.0 ± 0.0 | 0.0 ± 0.0 | 0.0 ± 0.0 |
| 26 | 0.0 ± 0.0 | 0.0 ± 0.0 | 38.2 ± 0.29 | 0.0 ± 0.0 | 0.0 ± 0.0 |
| 27 | 0.0 ± 0.0 | 0.0 ± 0.0 | 40.0 ± 0.0 | 0.0 ± 0.0 | 0.0 ± 0.0 |
| 28 | 0.0 ± 0.0 | 0.0 ± 0.0 | 0.0 ± 0.0 | 0.0 ± 0.0 | 0.0 ± 0.0 |
| 29 | 0.0 ± 0.0 | 0.0 ± 0.0 | 0.0 ± 0.0 | 0.0 ± 0.0 | 0.0 ± 0.0 |
| 30 | 0.0 ± 0.0 | 0.0 ± 0.0 | 0.0 ± 0.0 | 0.0 ± 0.0 | 0.0 ± 0.0 |

**Table S4.** P-values of pairwise comparisons of survival curves of adult emergence probability.

| **Comparison** | **Log-rank (Mantel-Cox) Test** | | |
| --- | --- | --- | --- |
|  | **χ2** | **df** | **p** |
| Control vs 25A | 306.5 | 1 | < 0.0001* |
| Control vs 50A | 420.9 | 1 | < 0.0001* |
| Control vs 75A | 448.9 | 1 | < 0.0001* |
| Control vs 100A | 448.9 | 1 | < 0.0001* |

*: Significance level p-value < 0.05

**Table S5.** Cumulative mortality rates of larvae fed on different diets using whole microalgae with different proportions from day 1 to day 30 are shown in Mean ± SD.

| Day | Mortality rate (Mean ± SD) | | | | |
| --- | --- | --- | --- | --- | --- |
|  | Control | 25A | 50A | 75A | 100A |
| 1 | 0.0 ± 0.0 | 0.0 ± 0.0 | 0.0 ± 0.0 | 0.0 ± 0.0 | 0.0 ± 0.0 |
| 2 | 0.0 ± 0.0 | 0.0 ± 0.0 | 0.0 ± 0.0 | 0.0 ± 0.0 | 0.0 ± 0.0 |
| 3 | 0.0 ± 0.0 | 0.0 ± 0.0 | 0.0 ± 0.0 | 0.0 ± 0.0 | 0.0 ± 0.0 |
| 4 | 0.0 ± 0.0 | 0.0 ± 0.0 | 0.0 ± 0.0 | 0.0 ± 0.0 | 0.0 ± 0.0 |
| 5 | 0.0 ± 0.0 | 0.0 ± 0.0 | 0.0 ± 0.0 | 5.2 ± 0.76 | 0.0 ± 0.0 |
| 6 | 0.0 ± 0.0 | 0.0 ± 0.0 | 0.0 ± 0.0 | 8.8 ± 0.76 | 0.0 ± 0.0 |
| 7 | 0.0 ± 0.0 | 0.0 ± 0.0 | 0.0 ± 0.0 | 13.8 ± 1.26 | 0.0 ± 0.0 |
| 8 | 0.0 ± 0.0 | 0.0 ± 0.0 | 0.0 ± 0.0 | 17.5 ± 0.5 | 0.0 ± 0.0 |
| 9 | 0.0 ± 0.0 | 0.0 ± 0.0 | 0.0 ± 0.0 | 20.3 ± 0.58 | 5.0 ± 0.5 |
| 10 | 0.0 ± 0.0 | 0.0 ± 0.0 | 0.0 ± 0.0 | 25.7 ± 1.15 | 7.5 ± 0.87 |
| 11 | 0.0 ± 0.0 | 0.0 ± 0.0 | 0.0 ± 0.0 | 30.5 ± 1.32 | 10.3 ± 0.58 |
| 12 | 0.0 ± 0.0 | 0.0 ± 0.0 | 0.0 ± 0.0 | 38.0 ± 2 | 17.7 ± 1.04 |
| 13 | 0.0 ± 0.0 | 0.0 ± 0.0 | 0.0 ± 0.0 | 45.7 ± 1.25 | 24.3 ± 0.29 |
| 14 | 0.0 ± 0.0 | 0.0 ± 0.0 | 0.0 ± 0.0 | 49.7 ± 0.58 | 31.8 ± 0.29 |
| 15 | 0.0 ± 0.0 | 0.0 ± 0.0 | 0.0 ± 0.0 | 53.3 ± 1.04 | 38.7 ± 0.58 |
| 16 | 0.0 ± 0.0 | 0.0 ± 0.0 | 0.0 ± 0.0 | 60.7 ± 0.76 | 45.7 ± 1.44 |
| 17 | 0.0 ± 0.0 | 0.0 ± 0.0 | 0.0 ± 0.0 | 65.2 ± 0.29 | 53.8 ± 1.61 |
| 18 | 0.0 ± 0.0 | 0.0 ± 0.0 | 1.0 ± 0.22 | 71.0 ± 0.5 | 60.8 ± 2.02 |
| 19 | 0.0 ± 0.0 | 0.0 ± 0.0 | 0.0 ± 0.0 | 74.7 ± 0.29 | 72.2 ± 1.26 |
| 20 | 0.0 ± 0.0 | 0.0 ± 0.0 | 1.5 ± 0.22 | 79.7 ± 0.29 | 85.3 ± 0.76 |
| 21 | 0.0 ± 0.0 | 0.0 ± 0.0 | 0.0 ± 0.0 | 85.3 ± 0.76 | 100.0 ± 0.0 |
| 22 | 0.0 ± 0.0 | 0.0 ± 0.0 | 2.0 ± 0.22 | 87.5 ± 0.0 | 0.0 ± 0.0 |
| 23 | 0.0 ± 0.0 | 0.0 ± 0.0 | 0.0 ± 0.0 | 88.7 ± 0.29 | 0.0 ± 0.0 |
| 24 | 0.0 ± 0.0 | 0.0 ± 0.0 | 0.0 ± 0.0 | 89.5 ± 0.0 | 0.0 ± 0.0 |
| 25 | 0.0 ± 0.0 | 0.0 ± 0.0 | 0.0 ± 0.0 | 0.0 ± 0.0 | 0.0 ± 0.0 |
| 26 | 0.0 ± 0.0 | 0.0 ± 0.0 | 0.0 ± 0.0 | 0.0 ± 0.0 | 0.0 ± 0.0 |
| 27 | 0.0 ± 0.0 | 0.0 ± 0.0 | 0.0 ± 0.0 | 0.0 ± 0.0 | 0.0 ± 0.0 |
| 28 | 0.0 ± 0.0 | 0.0 ± 0.0 | 0.0 ± 0.0 | 0.0 ± 0.0 | 0.0 ± 0.0 |
| 29 | 0.0 ± 0.0 | 0.0 ± 0.0 | 0.0 ± 0.0 | 0.0 ± 0.0 | 0.0 ± 0.0 |
| 30 | 0.0 ± 0.0 | 0.0 ± 0.0 | 0.0 ± 0.0 | 0.0 ± 0.0 | 0.0 ± 0.0 |

**Table S6.** P-values of pairwise comparisons of survival curves of mortality probability.

| **Comparison** | **Log-rank (Mantel-Cox) Test** | | |
| --- | --- | --- | --- |
|  | **χ2** | **df** | **p** |
| Control vs 25A | 0.000 | 1 | 0.9990 |
| Control vs 50A | 4.030 | 1 | 0.0447* |
| Control vs 75A | 364.5 | 1 | < 0.0001* |
| Control vs 100A | 455.8 | 1 | < 0.0001* |

*: Significance level p-value < 0.05

**Table S7.** Cumulative pupation rates of larvae fed on four different diets using disrupted cell materials (CD) and soluble fractions (SM) from day 1 to day 15 are shown in Mean ± SD.

| Day | Pupation rate (Mean ± SD) | | | |
| --- | --- | --- | --- | --- |
|  | Control | 62.5%WM+F | 62.5%CD+F | 62.5%SM+F |
| 1 | 0.0 ± 0.0 | 0.0 ± 0.0 | 0.0 ± 0.0 | 0.0 ± 0.0 |
| 2 | 0.0 ± 0.0 | 0.0 ± 0.0 | 0.0 ± 0.0 | 0.0 ± 0.0 |
| 3 | 0.0 ± 0.0 | 0.0 ± 0.0 | 0.0 ± 0.0 | 0.0 ± 0.0 |
| 4 | 0.0 ± 0.0 | 0.0 ± 0.0 | 0.0 ± 0.0 | 0.0 ± 0.0 |
| 5 | 16.7 ± 0.58 | 0.0 ± 0.0 | 0.0 ± 0.0 | 0.0 ± 0.0 |
| 6 | 31.7 ± 0.58 | 0.0 ± 0.0 | 0.0 ± 0.0 | 0.0 ± 0.0 |
| 7 | 48.3 ± 0.00 | 0.0 ± 0.0 | 0.0 ± 0.0 | 0.0 ± 0.0 |
| 8 | 68.3 ± 0.58 | 0.0 ± 0.0 | 0.0 ± 0.0 | 0.0 ± 0.0 |
| 9 | 86.7 ± 0.58 | 0.0 ± 0.0 | 0.0 ± 0.0 | 0.0 ± 0.0 |
| 10 | 100 ± 0.58 | 0.0 ± 0.0 | 0.0 ± 0.0 | 0.0 ± 0.0 |
| 11 | 0.0 ± 0.0 | 0.0 ± 0.0 | 0.0 ± 0.0 | 0.0 ± 0.0 |
| 12 | 0.0 ± 0.0 | 0.0 ± 0.0 | 0.0 ± 0.0 | 0.0 ± 0.0 |
| 13 | 0.0 ± 0.0 | 0.0 ± 0.0 | 0.0 ± 0.0 | 0.0 ± 0.0 |
| 14 | 0.0 ± 0.0 | 0.0 ± 0.0 | 0.0 ± 0.0 | 0.0 ± 0.0 |
| 15 | 0.0 ± 0.0 | 0.0 ± 0.0 | 0.0 ± 0.0 | 0.0 ± 0.0 |

**Table S8.** P-values of pairwise comparisons of survival curves of pupation probability.

| **Comparison** | **Log-rank (Mantel-Cox) Test** | | |
| --- | --- | --- | --- |
|  | **χ2** | **df** | **p** |
| Control vs WM+F | 183.4 | 1 | < 0.0001* |
| Control vs CD+F | 183.4 | 1 | < 0.0001* |
| Control vs SM+F | 183.4 | 1 | < 0.0001* |

*: Significance level p-value < 0.05

**Table S9.** Cumulative adult emergence rates of larvae fed on four different diets using disrupted cell materials (CD) and soluble fractions (SM) from day 1 to day 15 are shown in Mean ± SD.

| Day | Adult emergence rate (Mean ± SD) | | | |
| --- | --- | --- | --- | --- |
|  | Control | 62.5%WM+F | 62.5%CD+F | 62.5%SM+F |
| 1 | 0.0 ± 0.0 | 0.0 ± 0.0 | 0.0 ± 0.0 | 0.0 ± 0.0 |
| 2 | 0.0 ± 0.0 | 0.0 ± 0.0 | 0.0 ± 0.0 | 0.0 ± 0.0 |
| 3 | 0.0 ± 0.0 | 0.0 ± 0.0 | 0.0 ± 0.0 | 0.0 ± 0.0 |
| 4 | 0.0 ± 0.0 | 0.0 ± 0.0 | 0.0 ± 0.0 | 0.0 ± 0.0 |
| 5 | 0.0 ± 0.0 | 0.0 ± 0.0 | 0.0 ± 0.0 | 0.0 ± 0.0 |
| 6 | 0.0 ± 0.0 | 0.0 ± 0.0 | 0.0 ± 0.0 | 0.0 ± 0.0 |
| 7 | 16.7 ± 1.00 | 0.0 ± 0.0 | 0.0 ± 0.0 | 0.0 ± 0.0 |
| 8 | 35.0 ± 0.58 | 0.0 ± 0.0 | 0.0 ± 0.0 | 0.0 ± 0.0 |
| 9 | 50.0 ± 0.58 | 0.0 ± 0.0 | 0.0 ± 0.0 | 0.0 ± 0.0 |
| 10 | 68.3 ± 0.58 | 0.0 ± 0.0 | 0.0 ± 0.0 | 0.0 ± 0.0 |
| 11 | 86.7 ± 0.58 | 0.0 ± 0.0 | 0.0 ± 0.0 | 0.0 ± 0.0 |
| 12 | 100 ± 0.58 | 0.0 ± 0.0 | 0.0 ± 0.0 | 0.0 ± 0.0 |
| 13 | 0.0 ± 0.0 | 0.0 ± 0.0 | 0.0 ± 0.0 | 0.0 ± 0.0 |
| 14 | 0.0 ± 0.0 | 0.0 ± 0.0 | 0.0 ± 0.0 | 0.0 ± 0.0 |
| 15 | 0.0 ± 0.0 | 0.0 ± 0.0 | 0.0 ± 0.0 | 0.0 ± 0.0 |

**Table S10.** P-values of pairwise comparisons of survival curves of adult emergence probability.

| **Comparison** | **Log-rank (Mantel-Cox) Test** | | |
| --- | --- | --- | --- |
|  | **χ2** | **df** | **p** |
| Control vs WM+F | 187.4 | 1 | < 0.0001* |
| Control vs CD+F | 187.4 | 1 | < 0.0001* |
| Control vs SM+F | 187.4 | 1 | < 0.0001* |

*: Significance level p-value < 0.05

**Table S11.** Cumulative mortality rates of larvae fed on four different diets using disrupted cell materials (CD) and soluble fractions (SM) from day 1 to day 15 are shown in Mean ± SD.

| Day | Mortality rate (Mean ± SD) | | | |
| --- | --- | --- | --- | --- |
|  | Control | 62.5%WM+F | 62.5%CD+F | 62.5%SM+F |
| 1 | 0.0 ± 0.0 | 0.0 ± 0.0 | 0.0 ± 0.0 | 0.0 ± 0.0 |
| 2 | 0.0 ± 0.0 | 0.0 ± 0.0 | 0.0 ± 0.0 | 0.0 ± 0.0 |
| 3 | 0.0 ± 0.0 | 0.0 ± 0.0 | 0.0 ± 0.0 | 0.0 ± 0.0 |
| 4 | 0.0 ± 0.0 | 0.0 ± 0.0 | 0.0 ± 0.0 | 0.0 ± 0.0 |
| 5 | 0.0 ± 0.0 | 0.0 ± 0.0 | 0.0 ± 0.0 | 0.0 ± 0.0 |
| 6 | 0.0 ± 0.0 | 0.0 ± 0.0 | 0.0 ± 0.0 | 0.0 ± 0.0 |
| 7 | 0.0 ± 0.0 | 0.0 ± 0.0 | 0.0 ± 0.0 | 0.0 ± 0.0 |
| 8 | 0.0 ± 0.0 | 0.0 ± 0.0 | 0.0 ± 0.0 | 0.0 ± 0.0 |
| 9 | 0.0 ± 0.0 | 0.0 ± 0.0 | 0.0 ± 0.0 | 0.0 ± 0.0 |
| 10 | 0.0 ± 0.0 | 5.7 ± 0.58 | 0.0 ± 0.0 | 2.3 ± 1.15 |
| 11 | 0.0 ± 0.0 | 8.4 ± 0.58 | 6 ± 1 | 3.6 ± 1.15 |
| 12 | 0.0 ± 0.0 | 13.1 ± 0.58 | 8.7 ± 0.58 | 7.3 ± 0.58 |
| 13 | 0.0 ± 0.0 | 14.8 ± 0.58 | 13.4 ± 0.58 | 9 ± 0.58 |
| 14 | 0.0 ± 0.0 | 16.8 ± 1 | 15.1 ± 0.58 | 11.3 ± 1.15 |
| 15 | 0.0 ± 0.0 | 22.1 ± 0.58 | 15.8 ± 0.58 | 15 ± 0.58 |

**Table S12.** P-values of pairwise comparisons of survival curves of mortality probability.

| **Comparison** | **Log-rank (Mantel-Cox) Test** | | |
| --- | --- | --- | --- |
|  | **χ2** | **df** | **p** |
| Control vs WM+F | 18.4 | 1 | < 0.0001* |
| Control vs CD+F | 10.1 | 1 | < 0.0015* |
| Control vs SM+F | 9.03 | 1 | < 0.0027* |

*: Significance level p-value < 0.05
